# Supplementary material for: Elucidation of the low-expressing erythroid CR1 phenotype by bioinformatic mining of the GATA1-driven blood-group regulome
Source: Nat Commun. 2023 Aug 17;14:5001. doi: 10.1038/s41467-023-40708-w (PMC10435571; doi:10.1038/s41467-023-40708-w)
Supplement: Supplementary file 1 — Supplementary Information [file 41467_2023_40708_MOESM1_ESM.pdf]

Elucidation of the low-expressing erythroid CR1 phenotype by bioinformatic mining of the GATA1-driven blood-group regulome

Ping Chun Wu[1], Yan Quan Lee[1], Mattias Möller[1,2], Jill R. Storry[1,3], Martin L. Olsson[1,3]*

1. Division of Hematology and Transfusion Medicine, Department of Laboratory Medicine, Lund University, Lund, Sweden
2. Department of Clinical Genetics and Pathology, Office for Medical Services, Region Skåne, Lund, Sweden
3. Department of Clinical Immunology and Transfusion Medicine, Office for Medical Services, Region Skåne, Lund, Sweden

***Corresponding author**:
Prof. Martin L Olsson, M.D., Ph.D., BMC C14, Sölvegatan 19, SE-22184, Lund, Sweden;
Martin_L.Olsson@med.lu.se

Supplementary Tables

Supplementary Table 1 Genotyping of rs11117991 and rs2274567 in 100 healthy Swedish donors

The loci rs11117991 and rs2274567 are in complete LD in the Swedish cohort (n=100), where the genotype frequency for the homozygous major allele is 0.65, heterozygous is 0.32 and homozygous minor is 0.03.

rs2274567 / rs11117991	AA	AG	GG
**TT**	65	0	0
**TC**	0	32	0
**CC**	0	0	3

Supplementary Table 2 Genotyping of rs11117991 and rs2274567 in 396 healthy Thai donors

The loci rs11117991 and rs2274567 are in incomplete LD in the Thai cohort. A total of 11 out of 396 samples carried the third haplotype of the two SNVs, which was not observed in the Swedish cohort. Respective genotype frequencies out of 396 are shown in parentheses.

**rs2274567*** / **rs11117991**	**AA**	**AG**	**GG**	
**TT**	108(27.3%)	7 (1.8%)	0	115 (29%)
**TC**	0	191(48.2%)	4 (1%)	195 (49.2%)
**CC**	0	0	86(21.7%)	86 (21.7%)
	108 (27.3%)	198 (50%)	90 (22.7%)	

*Polymerase chain reaction restriction fragment length polymorphism (PCR-RFLP) showed that HindIII restriction site rs11118133A>T was in complete concordance with the rs2274567A>G genotyping result in the Thai cohort.

Supplementary Figures

	rs11117991	rs2274567	rs3737002	rs41274768	rs17047660	rs17047661	rs4844609	rs6691117	Haplotype frequency LDlink-1000 Genomes GRCh38					
	T/C	A/G	C/T	G/A	A/G	A/G	T/A	A/G	EUR	EAS	SAS	AFR	AMR	**ALL**
Haplotype 1	T	A	C	G	A	A	T	A	0.4662	0.3442	0.1892	0.0826	0.255	**0.2571**
Haplotype 2	T	A	T	G	A	A	T	A	0.2853	0.3115	0.3088	0.0447	0.3602	**0.2421**
Haplotype 3	C	G	C	G	A	A	T	G	0.1799	0.2956	0.4264	0.0182	0.2205	**0.2143**
Haplotype 4	T	A	C	G	A	G	T	G	0.005	0	0	0.3742	0.0245	**0.1031**
Haplotype 5	T	A	C	G	G	G	T	G	0.003	0	0	0.2697	0.0245	**0.0751**
Haplotype 6	T	G	C	G	A	A	T	G	0	0.0308	0.0644	0.1341	0.0476	**0.0607**
Haplotype 7	T	G	C	G	A	G	T	G	0.001	0	0	0.0652	0.0029	**0.0178**
Haplotype 8	T	A	C	A	A	A	T	G	0.0338	0.002	0.0031	0.0015	0.036	**0.0132**
Haplotype 9	T	A	T	G	A	A	T	G	0.002	0.0139	0	0	0.0173	**0.0056**
Haplotype 10	T	A	C	G	A	A	A	A	0.0199	0	0.001	0	0.0058	**0.005**
Haplotype 11	T	A	C	G	A	A	T	G	0	0.001	0	0.0083	0.0029	**0.0028**
Haplotype 12	C	A	C	G	A	A	T	A	0.002	0	0.001	0	0.0014	**0.0008**
Haplotype 13	C	G	T	G	A	A	T	A	0	0.001	0.0031	0		**0.0008**
Haplotype 14	C	A	C	G	A	A	T	G	0.001	0	0.001	0	0	**0.0004**
Haplotype 15	C	A	T	G	A	A	T	A	0	0	0.001	0	0.0014	**0.0004**
Haplotype 16	C	G	C	G	A	A	T	A	0.001	0	0.001	0	0	**0.0004**
Haplotype 17	T	G	C	G	A	A	T	A	0	0	0	0.0008	0	**0.0002**
Haplotype 18	T	G	C	G	G	G	T	G	0	0	0	0.0008	0	**0.0002**

Supplementary Figure 1: Haplotype frequencies of rs11117991, rs2274567 and loci of the Knops antigens on LDlink.

Haplotype frequencies as reported in the 1000 Genomes project were obtained from LDlink by LDhap. The loci encoding the Knops antigens (in parentheses) are: rs2274567 (DACY/YCAD), rs3737002 (Yk^a), rs41274768 (Kn^a/Kn^b), rs17047660 (McC^a/McC^b), rs17047661 (Sl1,Sl^a/Sl2,Vil), rs4844609 (Sl3), rs6691117 (KCAM/KDAS). EUR: European, EAS: East Asian, SAS: South Asian, AFR: African, AMR: Ad Mixed American.

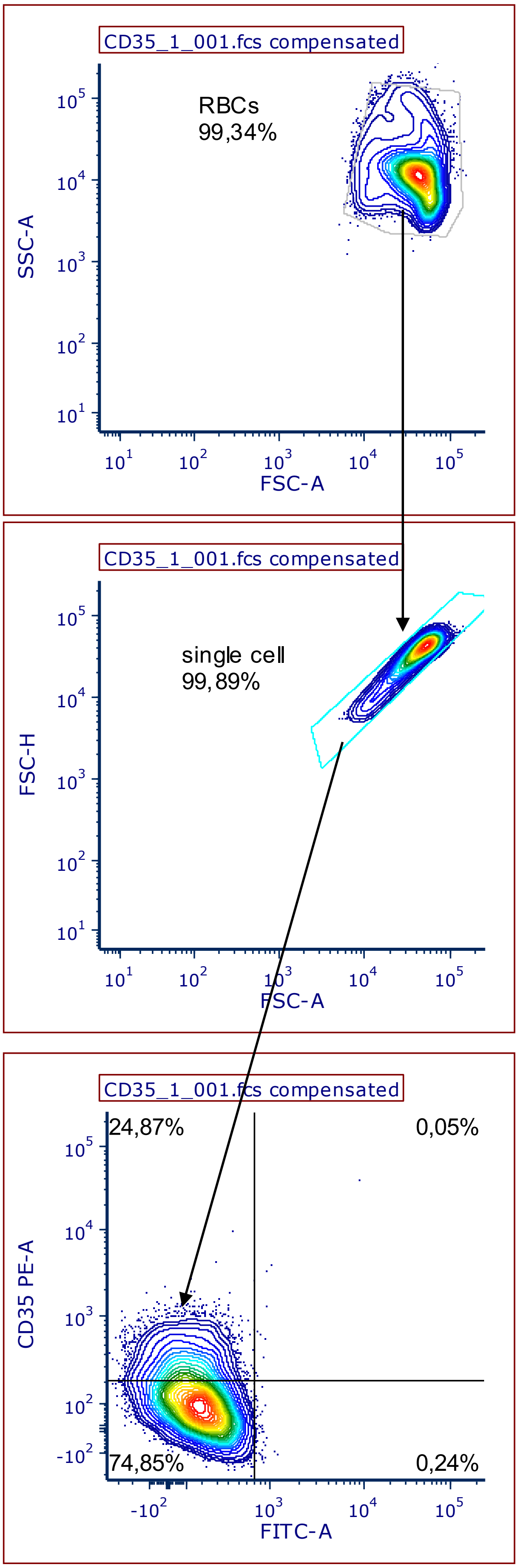

Supplementary Figure 2: The gating strategy used for flow cytometry.

Supplementary Note

Supplementary information about sequencing of samples with low CR1 expression

We sequenced a 952-bp fragment of the *CR1* region surrounding and including motifs 1 and 2 of seven samples carrying the rs11117991:T/T genotype but expressing lower CR1 in Fig. 5. The sequenced fragment was amplified with the primers mentioned in Suppl. Data 3 for luciferase assay plasmid construction. In total, seven samples (5 Swedish and 2 Thai) were tested:

1. Three samples had no deviations from the *CR1* reference sequence (NG_007481), although all samples carried rs10779311:C. However, its frequency in gnomAD is 0.9855.
2. One Swedish sample was heterozygous for rs61822967:G>A, which has a frequency of 0.2526 according to gnomAD.
3. Three other samples (two Swedish sample and one Thai) were heterozygous for rs12043913:G>T at 0.2512.
4. One (Thai) of the latter samples was also heterozygous for rs147061134:A>C, a SNV with a frequency of 0.0125 according to the 1000 Genomes project and 0.0248-0.0316 in Asian populations (but not found in gnomAD).
5. None of the above SNVs disrupt or are found adjacent to the two GATA1 motifs 1 and 2, nor did they interfere with binding sites for other key erythroid transcription factors.

In conclusion, we did not reveal any plausible explanation underlying the low expression in the rs11117991:T/T genotype group. The sequencing data generated in this study have been deposited in the European Nucleotide Archive (ENA) under the project code PRJEB64594.
