# Supplementary material for: Elucidation of the low-expressing erythroid CR1 phenotype by bioinformatic mining of the GATA1-driven blood-group regulome
Source: Nat Commun. 2023 Aug 17;14:5001. doi: 10.1038/s41467-023-40708-w (PMC10435571; doi:10.1038/s41467-023-40708-w)
Supplement: Supplementary file 3 — Description of Additional Supplementary Files [file 41467_2023_40708_MOESM3_ESM.pdf]

Description of Additional Supplementary Files

File Name: Supplementary Data 1
Description: A total of 193 GATA1-binding sites predicted from ChIP-seq analysis in blood group genes using GRCh38/hg38 as reference assembly. This list has been filtered to peaks with one or more overlaps after intersecting the datasets.
*Encoding the P1PK blood group system (P was the old system name prior to 2010).

File Name: Supplementary Data 2
Description: ChIP-seq datasets analyzed in this study.
*The experiments were merged using MACS for joint peak calling before intersecting peaks with the reference dataset as they are from the same biosample.

File Name: Supplementary Data 3
Description: Probe and primer sequences used in this study.
