# Supplementary material for: Elucidation of the low-expressing erythroid CR1 phenotype by bioinformatic mining of the GATA1-driven blood-group regulome
Source: Nat Commun. 2023 Aug 17;14:5001. doi: 10.1038/s41467-023-40708-w (PMC10435571; doi:10.1038/s41467-023-40708-w)
Supplement: Supplementary file 7 — Reporting Summary [file 41467_2023_40708_MOESM7_ESM.pdf]

nature portfolio

Corresponding author(s): Prof. Martin L. Olsson, M.D., Ph.D.

Last updated by author(s): Jun 27, 2023

Reporting Summary

Nature Portfolio wishes to improve the reproducibility of the work that we publish. This form provides structure for consistency and transparency in reporting. For further information on Nature Portfolio policies, see our Editorial Policies and the Editorial Policy Checklist.

Statistics

For all statistical analyses, confirm that the following items are present in the figure legend, table legend, main text, or Methods section.

n/a	Confirmed	
☐	☒	The exact sample size (*n*) for each experimental group/condition, given as a discrete number and unit of measurement
☐	☒	A statement on whether measurements were taken from distinct samples or whether the same sample was measured repeatedly
☐	☒	The statistical test(s) used AND whether they are one- or two-sided<br>*Only common tests should be described solely by name; describe more complex techniques in the Methods section.*
☐	☒	A description of all covariates tested
☐	☒	A description of any assumptions or corrections, such as tests of normality and adjustment for multiple comparisons
☐	☒	A full description of the statistical parameters including central tendency (e.g. means) or other basic estimates (e.g. regression coefficient) AND variation (e.g. standard deviation) or associated estimates of uncertainty (e.g. confidence intervals)
☐	☒	For null hypothesis testing, the test statistic (e.g. *F*, *t*, *r*) with confidence intervals, effect sizes, degrees of freedom and *P* value noted<br>*Give P values as exact values whenever suitable.*
☒	☐	For Bayesian analysis, information on the choice of priors and Markov chain Monte Carlo settings
☒	☐	For hierarchical and complex designs, identification of the appropriate level for tests and full reporting of outcomes
☒	☐	Estimates of effect sizes (e.g. Cohen's *d*, Pearson's *r*), indicating how they were calculated

Our web collection on statistics for biologists contains articles on many of the points above.

Software and code

Policy information about availability of computer code

Data collection

1) Flow cytometry: BD FACSDiva v 8.0.1 (for BD FACSCanto II) or CellStream Analysis v 1.4.72 (for Amnis CellStream)
2) qPCR and genotyping: QuantStudio Design & Analysis v 1.5.1
3) GloMax Discover v 3.0
4) ChemiDoc Touch Image System v2.3.0.07

Data analysis

1) nf-core analysis pipeline (v1.0.0, https://github.com/nf-core/chipseq)
2) MACS2 (v2.1.0.20150731-Python-2.7.11)
3) BEDtools (v2.26.0)
4) ChIPseeker (v1.22.1)
5) RStudio (v3.6.3) with ChIPpeakAnno package (v3.20.1)
6) MEME Suite (v5.0.4)
7) JASPAR (v2020)
8) gnomAD (v3.1.2)
9) Ensembl GRCh38 (Last accessed August 1st, 2022)
10) Image Lab (Bio-Rad, v6.0.1)
11) Flow cytometry: FCS Express 6 Flow Cytometry-RUO (v6.06.0042)
12) qPCR: QuantStudio Design & Analysis (v1.5.1)
13) LDlink (v5.3)
14) All scripts used available at https://github.com/ILM-MLOlab/ChIP-seq-GATA1 or https://zenodo.org/badge/latestdoi/501283332.
15) OpenRefine (v3.5.2)
16) GraphPad Prism (v9.3.1)

For manuscripts utilizing custom algorithms or software that are central to the research but not yet described in published literature, software must be made available to editors and reviewers. We strongly encourage code deposition in a community repository (e.g. GitHub). See the Nature Portfolio guidelines for submitting code & software for further information.

Data

Policy information about availability of data

All manuscripts must include a data availability statement. This statement should provide the following information, where applicable:

- Accession codes, unique identifiers, or web links for publicly available datasets
- A description of any restrictions on data availability
- For clinical datasets or third party data, please ensure that the statement adheres to our policy

All data generated and/or analyzed during the current study are either included in this published article (and its supplementary information files), or are available from the corresponding author upon reasonable request.

Human research participants

Policy information about studies involving human research participants and Sex and Gender in Research.

Reporting on sex and gender

We included blood samples from blood donors of both sexes in this study. For the Swedish cohort of randomly collected blood samples obtained as anonymized waste products following routine blood donation, no information about donor sex, gender or age is available. For the Thai cohort, the ethics approval and consent included information about the donors' sex (42% women, 58% men). The average percentage in the Southern region of Sweden (Skåne) is known (43% women, 57% men) and similar to that in the Thai cohort.
The CR1 gene is carried on chromosome 1 and GATA1 is encoded on the X chromosome but expression levels in both men and women can be considered similar due to the X chromosome inactivation in women. Thus, our research question was not to find any possibly sex-dependent differences in expression of CR1 but we have no data to indicate that expression levels would be sex-dependent. Our findings on the genetic basis of the Helgeson phenotype can be applied to both men and women.

Population characteristics

The two cohorts used for in vitro validation of the hypothesis generated in silico are the following:
1) Apparently healthy blood donors from southern Sweden (Region Skåne). Donation is typically allowed between 18 and 65 years of age and body weight must be >50 kg.
2) Apparently healthy blood donors from two regions in Thailand (Lampang in the north and Saraburi in central Thailand). Donation is typically allowed between 17 and 70 years of age and body weight must be >45 kg.

Recruitment

No participants were actively recruited to take part in this study. Instead, reference material previously collected as part of routine, random blood donation at blood centers in Thailand for blood group research on anonymized samples was used also for CR1/KN blood group studies (within the permissions obtained), without prior knowledge about the donors' CR1 expression levels. The Swedish samples were collected as described below.

Ethics oversight

For the Thai cohort of healthy blood donors, anticoagulated blood samples were obtained from the Lampang Hospital and Saraburi Hospital, Thailand, following ethics approval (UP-HEC 2/024/59) and informed consent. The approval was given by the University of Phayao Human Ethics Committee, Thailand. According to the Swedish research law, using waste/excess fully anonymized and/or pooled biological material does not require ethics approval. Thus, no particular approval was required for this study, apart from the permission quoted above, and from the Dept. of Clinical Immunology and Transfusion Medicine to obtain these anonymized blood samples from waste material (ref. no. 2018:22 and 2020:16, as referenced in the manuscript).

Note that full information on the approval of the study protocol must also be provided in the manuscript.

Field-specific reporting

Please select the one below that is the best fit for your research. If you are not sure, read the appropriate sections before making your selection.

☒ Life sciences ☐ Behavioural & social sciences ☐ Ecological, evolutionary & environmental sciences

For a reference copy of the document with all sections, see nature.com/documents/nr-reporting-summary-flat.pdf

Life sciences study design

All studies must disclose on these points even when the disclosure is negative.

Sample size	The two cohorts used here (as described above) constitute reference collections of random blood donors (n=100 Swedish donors and n=396 Thai donors) used for blood group research in our laboratory. No formal size calculation was performed. However, the available samples were deemed sufficient based on the allele frequency of interest.
Data exclusions	No data were excluded.
Replication	For EMSA: Three independent experiments were performed for each selected target predicted by the ChIP-seq data. All attempts at replication were successful.<br>For Luciferase assay: All samples were run in technical triplicates and experiments were performed three times.<br>For gene expression and genotyping assays, the technical replicates were done in the same run and were successful.<br>For flow cytometry the experiments were done once with at least 10,000 events collected for each sample.<br>For western blots, at least one successful replicate was performed.
Randomization	Donor cohorts consisted of unselected, random donors at the blood centres. Randomization of donors was not relevant for this study because the aim was to investigate a random sample from the Swedish and Thai population.
Blinding	The design of the study did not call for or include blinding but in a fact all assays were performed without prior knowledge of the genotype and/or phenotypes of the respective donors. The exception was the western blots for which the samples were selected to compare between three different genotypes.

Reporting for specific materials, systems and methods

We require information from authors about some types of materials, experimental systems and methods used in many studies. Here, indicate whether each material, system or method listed is relevant to your study. If you are not sure if a list item applies to your research, read the appropriate section before selecting a response.

Materials & experimental systems

n/a	Involved in the study	
☐	☒	Antibodies
☐	☒	Eukaryotic cell lines
☒	☐	Palaeontology and archaeology
☒	☐	Animals and other organisms
☒	☐	Clinical data
☒	☐	Dual use research of concern

Methods

n/a	Involved in the study	
☒	☐	ChIP-seq
☐	☒	Flow cytometry
☒	☐	MRI-based neuroimaging

Antibodies

Antibodies used	1) EMSA: rabbit anti-GATA1 pAb, Active Motif #61535<br>2) Immunoblot: mouse anti-human CD35 clone E11, Bio-Rad #MCA554GA and goat anti-mouse IgG HRP pAb, Bio-Rad #1706516<br>3) Flow cytometry: PE Mouse Anti-Human CD35 clone E11, BD Pharmingen #559872
Validation	1) Anti-GATA1 (used for EMSA) was validated by Active Motif for ChIP-seq in K562 cells.<br>2) Anti-CD35 clone E11 previously reported to be used for Western blot. Lach-Trifilieff et al. The Journal of Immunology 162, 7549–7554 (1999).<br>3) Epitope of anti-CD35 clone 11 mapped in CHO cells expressing CR1 mutants. Also validated flow cytometry here. Nickells, M. et al. Clinical and Experimental Immunology 112, 27–33 (1998).

Eukaryotic cell lines

Policy information about cell lines and Sex and Gender in Research

Cell line source(s)	K562 (female) and HEL (male), both obtained from department's archive of frozen cell lines.
Authentication	Neither of the cell lines were authenticated. Neither of the cell lines used in this study were reported in the "Register of Misidentified Cell Lines".
Mycoplasma contamination	Cell lines were not tested for Mycoplasma contamination during the study.
Commonly misidentified lines (See ICLAC register)	None

Flow Cytometry

Plots

Confirm that:

☒ The axis labels state the marker and fluorochrome used (e.g. CD4-FITC).

☒ The axis scales are clearly visible. Include numbers along axes only for bottom left plot of group (a 'group' is an analysis of identical markers).

☒ All plots are contour plots with outliers or pseudocolor plots.

☒ A numerical value for number of cells or percentage (with statistics) is provided.

Methodology

Sample preparation	3% suspensions of fresh or glycerol-frozen/thawed erythrocytes were prepared from peripheral blood following three washes in phosphate-buffered saline.
Instrument	1) BD FACSCanto II<br>2) Amnis CellStream
Software	1) For FACSCanto II: BD FACSDiva (v8.0.1)<br>2) For CellStream: CellStream Analysis (v1.4.72)<br>3) For analysis of data from both flow cytometers: FCS Express 6 Flow Cytometry-RUO (v6.06.0042)
Cell population abundance	No sorting performed. No FACS plots included in the manuscript.
Gating strategy	Flow cytometry experiments were performed on washed erythrocytes (constituting >99% of all cells present) from peripheral blood samples. The red blood cells were gated using the FSC-A and SSC-A for the cell population excluding the debris. Thus, the blood group typing was performed on all gated erythrocytes in the sample.

☒ Tick this box to confirm that a figure exemplifying the gating strategy is provided in the Supplementary Information.
